# ASHE December 2021: Reflections on our first year with an eye on the future

**DOI:** 10.1017/ash.2021.262

**Published:** 2022-03-30

**Authors:** Gonzalo Bearman, Anucha Apisarnthanarak, Alexandre R. Marra, Kelly Matson, Priya Nori, Kari A. Simonsen, Pranavi Sreeramoju, Lindsay MacMurray

**Affiliations:** 1 Division of Infectious Diseases, Virginia Commonwealth University, Richmond, Virginia, United States; 2 Division of Infectious Diseases, Faculty of Medicine, Thammasat University, Prathum Thani, Thailand; 3 Department of Internal Medicine, University of Iowa Carver College of Medicine, Iowa City, Iowa, United States; 4 Instituto Israelita de Ensino e Pesquisa Albert Einstein, Hospital Israelita Albert Einstein, São Paulo, Brazil; 5 Center for Access & Delivery Research & Evaluation (CADRE), Iowa City Veterans’ Affairs Health Care System, Iowa City, Iowa, United States; 6 The University of Rhode Island, College of Pharmacy, Kingston, Rhode Island, United States; 7 Division of Infectious Diseases, Department of Medicine, Montefiore Medical Center, Albert Einstein College of Medicine, Bronx, New York, United States; 8 Department of Pediatrics, University of Nebraska Medical Center, Omaha, Nebraska, United States; 9 Children’s Hospital and Medical Center, Omaha, Nebraska, United States; 10 Division of Medicine–Infectious Diseases, UT Southwestern Medical Center, Dallas, Texas, United States; 11 The Society for Healthcare Epidemiology of America, Arlington, Virginia, United States

As 2021 draws to a close, we pause to reflect on this year’s launch of *Antimicrobial Stewardship and Healthcare Epidemiology*—ASHE. The journal is committed to the rapid dissemination of high-quality, timely, evolving science and implementation knowledge in the field of antimicrobial stewardship and healthcare epidemiology. With an Open Access model, it serves professionals all over the world, regardless of the income status of their country. We are committed to diversity in professional background, nationality, and sex across the editorial team, such that journal leadership reflects not only the membership of the Society for Healthcare Epidemiology of America but also the antimicrobial stewardship and healthcare epidemiology community at large.

Although not yet a full year of age, ASHE was officially launched with an inaugural editorial publication on June 23, 2021. Since then, we have published 22 original investigations, 13 concise communications, 10 research briefs, 10 invited reviews, 3 commentaries, and 4 letters to the editor across a diversity of perspectives in healthcare epidemiology and antimicrobial stewardship. In addition, we published the proceedings from SHEA Spring 2021. Last, our goal to include colleagues in low- and middle-income countries (LMICs) has been affirmed by recently published manuscripts from Africa, Asia, Europe, North America, and South America.

The most immediate measures of success include the 167 submissions, 14,208 article downloads, 443 Altmetric mentions, and 3 citations. As a new journal, we have exceeded expectations on multiple fronts, including the number of papers published and the overall impact of content published.

We deliberately sought an editorial team that represented the diversity and background of not only SHEA membership but also the fields of antimicrobial stewardship and healthcare epidemiology. The team includes pharmacists, pediatricians, and nurses in a variety of geographic locations and healthcare settings. We anticipate a growing diversity of voices and perspectives in ASHE. Because ASHE is an Open Access journal, the version of record of every article is made freely available to all immediately upon publication, with additional usage rights. Although most authors have access to funding to cover article processing charges or are at institutions that have signed a Read & Publish agreement with Cambridge University Press, we strongly believe that there should be no financial barrier to publication in ASHE. Thus, we have instituted a comprehensive waiver policy. We have an automatic waiver scheme for World Bank–designated LMICs and for any other authors who are unable to pay the article processing charges in full or in part; these authors can request a discount or waiver from Cambridge University Press. In addition, SHEA members are entitled to a 50% discount for the article processing charges.

Importantly, the successful launch of ASHE reflects multiple elements, among which the support of SHEA leadership has been hugely influential. The Cambridge University Press partnership, collaborations with ICHE and the SHEA Publications Committee, and the overwhelming enthusiasm and content contributions from SHEA membership and the healthcare epidemiology and antimicrobial stewardship community at large have enabled the success of ASHE in its inaugural year.

We look forward to the coming year with hope, enthusiasm, and greater aspirations. We invite you to contribute your best work related to antimicrobial stewardship, infection prevention, occupational health related to infections, and stewardship related to infectious diseases diagnostic tests, particularly as they relate to healthcare-associated infections and antimicrobial resistance. In addition to the traditional types of articles, such as original articles, review articles, concise communications, research briefs, and letters to editor, we invite you to contribute 2 new article types. One is “reflections in healthcare epidemiology,” which share interesting cases and vignettes encountered in professional practice along with leadership takeaways. The second new article type is “careers,” which is primarily oriented toward learners and early career professionals and aims to support, guide, and promote careers related to healthcare epidemiology. Please check out our journal website for more information.

We would like to reiterate that we are deeply grateful for all the contributors, reviewers, and readers who continue to grow the scope, relevance, and impact of ASHE as an influential Open Access journal. We look forward to greater engagement with you in the coming year.

